# Bringing light to the reading room

**DOI:** 10.3389/fradi.2026.1821486

**Published:** 2026-06-12

**Authors:** Christine Joyce, Mohammad Adam Kassar, Alexandria Knecht, Melissa Jung, Joe Joseph, Justin McCloskey, Jarunee Intrapiromkul, Dhairya A. Lakhani, Tyler McGaughey

**Affiliations:** 1 Department of Radiology, West Virginia University, Morgantown, WV, United States; 2 Department of Neuroradiology, West Virginia University, Morgantown, WV, United States; 3 West Virginia Clinical and Translational Sciences Institute, Morgantown, WV, United States

**Keywords:** ambient light, brain hemorrhage, computed tomgraphy (CT), diagnostic accuracy, monitor

## Abstract

**Background:**

According to the American College of Radiology (ACR) Technical Standard Guidelines, there is a recommended illuminance level for reading rooms set between 25 and 75 lux. However, these guidelines are based on studies completed in 2007–2009, which predate significant advancements in display technology. No recent study has re-evaluated these lighting conditions, and more specifically, no prior studies have examined the effects of ambient lighting on the detection of intracranial hemorrhage on non-contrast brain CT.

**Objective:**

To determine if higher ambient lighting levels (> 400 lux) significantly effects the diagnostic accuracy of board-certified neuroradiologists in detecting intracranial hemorrhage on brain CT, compared with standard low-light reading room conditions (20–70 lux).

**Methods:**

Forty-two standard-of-care non-contrast head CT scans (axial acquisitions with coronal/sagittal reformats) from a tertiary-care hospital were selected by two independent neuroradiologists. These neuroradiologists established the presence or absence of intracranial hemorrhage. Four additional board-certified neuroradiologists independently interpreted all cases twice: first in a brightly lit environment (> 400 lux, measured at desktop level) and, after a > 6-month washout period to minimize recognition memory bias, in a standard reading room (20–70 lux). Interpretations were performed on calibrated Barco MDNC-3421 displays using the institutional Fuji PACS. Sensitivity, specificity, and accuracy were calculated for each condition and compared using paired two-tailed t-tests with Bonferroni correction for multiple comparisons.

**Results:**

No statistically significant differences were found between bright and dark conditions for sensitivity (0.974 vs. 0.974, *p* = 1.00), specificity (0.978 vs. 1.0, *p* = 0.49 adjusted), or overall accuracy (0.976 vs. 0.988, *p* = 1.00 adjusted).

**Conclusion:**

Increased ambient lighting levels (> 400 lux) do not significantly affect the ability of board-certified neuroradiologists to accurately detect intracranial hemorrhage on modern high-resolution displays. These findings suggest that current guidelines should be re-evaluated with recent improvements in technology. Larger multi-center studies are warranted to confirm these results across additional pathologies and modalities.

## Introduction

Traditionally, it has been believed that radiology reading rooms should minimize ambient lighting to enhance image contrast for the diagnosis of subtle findings. However, with the transition from hard-copy films to high-resolution display monitors, these beliefs are becoming outdated. Only a few studies have explored the implications of ambient lighting in reading rooms, the most recent of which was conducted in 2020. This study revealed insufficient data to determine whether dark room conditions are necessary for accurate image diagnosis (1).

Despite advancements in modern technology, no definitive studies have established the need for dark rooms in radiology. Moreover, the existing literature review indicates that no research has been conducted on evaluating brain CTs in various lighting environments within reading rooms. Ambient lighting is quantified in the international system of units, “lux,” a measurement for light that falls on a surface. According to the American College of Radiology (ACR) Technical Standard Guidelines (2), the recommended illuminance level for reading rooms is between 25 and 75 lux, which helps to prevent eye fatigue and minimize reflections on workstation displays (2). It is also advised that ambient light fixtures be indirect or backlit and equipped with dimmers, avoiding fluorescent lighting and overhead lights (3).

Despite these recommendations, no recent studies have supported these current guidelines, taking into consideration the advancements in display technology. These current standards were based on studies published in 2007 and 2009, almost 20 years prior (2). Additionally, the guidelines are quite general and do not address the specific needs of different radiology subspecialties. Therefore, the objective of this study is to demonstrate that, due to advancements in digital technology, ambient light does not significantly affect the accurate diagnosis of brain hemorrhage on brain CTs.

## Methods

All study-related procedures were approved by the Institutional Review Board (IRB) #2407009406 AAHRPP accredited IRB.

### Test case selection

The head CTs selected for this case were chosen by two independent board-certified neuroradiologists. These were acquired as axial images of the brain with reformatted coronal and sagittal images without administration of intravenous contrast. A sample of 42 standard-of-care head CT scans were selected from a tertiary-level hospital in West Virginia. 18 of the 42 selected cases were positive for a brain hemorrhage, as identified by two separate board-certified radiologists. Both reviewers were instructed to review the scans for the presence of intracranial hemorrhage under different lighting conditions to establish the ground truth key. Intracranial hemorrhage was chosen as the pathology of choice for this study since it is a common pathology, a frequently ordered exam, and carries a high risk of adverse outcomes if not identified. This pathology was also chosen due to this being a relatively objective finding. Many of the more subtle intracranial neuroradiology findings on a CT brain can be somewhat subjective, leading to a high inter-reader variability.

### Participant reviewer methods

Four different board-certified neuroradiologists from the two case selectors were selected as test participant reviewers. The reviewers were instructed to read the selected scans in a well-illuminated environment (> 400 lux). Lux was measured at the desktop level via an Elitech digital illuminance meter. Scans were read using the standard Fuji PACs environment on Barco MDNC-3421 monitors with the standard calibration settings. Readers were instructed to indicate hemorrhage as positive or negative.

A study was published in 2011 about the “memory effect” for repeated radiologic observations. In this study, it was shown that when the same images were presented for a second time after a short period, there was clear recognition memory (4). Therefore, we allowed for a washout period of greater than six months and then had the neuroradiologist review the images for a second time at a standard reading room of 20–70 lux. This was done to allow for a washout period and to avoid recognition memory effects.

### Data analysis methods

A dependent two-tailed t-Test was done to compare sensitivity, specificity, and accuracy between the groups. In our investigation, the unit of analysis for inferential statistics was the expert reader, not the individual case. Summary performance metrics (sensitivity, specificity, and accuracy) between conditions were calculated for each reader. These reader-based estimates were then treated as continuous variables, and paired two-tailed t-tests were used to compare performance between conditions. We corrected for multiple comparisons via a Bonferroni adjustment. There were no significant differences between conditions. However, it is important to note that experimental light conditions are slightly less specific than the control dark, but it is not a significant difference. A Fleiss Kappa test was conducted for inter-reader reliability.

## Results

Our study expands upon the findings of previous research by evaluating a broader range of ambient lighting conditions across a critical imaging modality. The study design incorporates a more diverse array of lighting conditions than those explored in prior studies, enabling us to better reflect the complexities of modern radiological environments. There was a near-perfect degree of agreement between all readers via a Fleiss Kappa test, with a kappa value of 0.90 in both experimental and control conditions. The descriptive proportions show an identical sensitivity between conditions (0.974, 95% CI 0.900–0.995). While specificity and accuracy were slightly higher in dark conditions (specificity 1.000 vs. 0.978; accuracy 0.988 vs. 0.976), it is important to note that there is a large overlap in the confidence intervals. These values are shown in a tabular form in Table 1.

**Table 1:** Descriptive statistics for each condition.

Condition	Sensitivity	Sens 95% CI Lower	Sens 95% CI Upper	Specificity	Spec95% CI Lower	Spec 95% CI Upper	Accuracy	Acc 95% CI Lower	Acc 95% CI Upper
Light	0.974	0.900	0.995	0.978	0.916	0.996	0.976	0.936	0.992
Dark	0.974	0.900	0.995	1.000	0.950	1.000	0.988	0.953	0.998

We used a dependent two-tailed t-Test to determine differences in the sensitivity, specificity, and accuracy between the light and dark conditions, also shown in Table 2. Sensitivity was identical between conditions s (*Δ* = 0.000, 95% CI −0.137–0.137, *p* = 1.000). Specificity was slightly higher under dark conditions, though not significant with notable overlap in confidence intervals (*Δ* = −0.022, 95% CI −0.091–0.047, *p* = 0.39). Lastly, accuracy was also similar between conditions (*Δ* = −0.012, 95% CI −0.084–0.061, *p* = 0.64). It's important to note that all confidence intervals span zero, indicating there is no meaningful difference between conditions by any metric. These results further support the idea that the current guidelines for reading conditions should be re-evaluated.

**Table 2:** Statistical comparisons between conditions.

Metric	Mean Light	Mean Dark	Mean Diff	t Stat	DF	*p* value	95% CI Lower	95% CI Upper	*p* value adj
Sensitivity	0.974	0.974	0.000	0.000	3.000	1.000	−0.137	0.137	1.000
Specificity	0.978	1.000	−0.022	−1.000	3.000	0.391	−0.091	0.047	1.000
Accuracy	0.976	0.988	−0.012	−0.522	3.000	0.638	−0.084	0.061	1.000

## Discussion

Ambient lighting plays a crucial role in the diagnostic accuracy of radiological imaging, particularly in the detection of acute neuroradiological abnormalities (7, 8). Image quality and contrast are directly influenced by lighting conditions, and proper ambient lighting is essential for minimizing eye strain and maximizing the radiologist's ability to interpret subtle findings (10, 11). Current ACR guidelines recommend reading room illuminance of 25–75 lux (2). These guidelines are based on studies published in 2008 and 2009 (2). Of note, radiology transitioned from film to digital imaging in the late 1990s and early 2000s, less than 10 years prior to the aforementioned studies being published (2, 13). With today's rapid advancements in imaging technology and display systems, these guidelines may no longer reflect the current needs of clinical practice.

Previous studies, such as those by Pollard et al., have explored the effects of increased ambient lighting on diagnostic accuracy for chest radiographs and mammograms (9, 12). Their research demonstrated that modest increases in ambient lighting (ranging from 1 lux to 50 lux) did not significantly impact the detection of abnormalities, including nodules on chest radiographs and lesions on mammograms (5, 6). That paper's findings suggest that controlled lighting conditions may reduce eye strain without compromising diagnostic performance. Again, it is important to note that these studies were conducted almost 20 years ago, and technological advancements in both imaging equipment and display systems have since occurred, potentially altering the dynamics between ambient lighting and diagnostic accuracy.

Of particular concern is the lack of research specifically investigating the impact of ambient lighting on brain CT evaluations. Although there have been studies on various radiological modalities, no research has directly addressed how lighting conditions influence the detection of acute neurological findings in brain CTs. Given the critical nature of timely and accurate diagnoses in neurology, this gap in the literature warrants further exploration.

Looking forward, there are several potential directions for future research. A key limitation of our investigation was the small number of readers (*n* = 4), which limits the generalizability of these findings. The limited number of readers increased the risk of Type II error, but the small observed effect sizes with confidence intervals centered near zero support that there was no meaningful difference in the precision of this investigation. Future research should aim to include a larger cohort to bolster statistical power and reliability. Expanding the study to include a broader range of clinical settings, including both academic and community-based radiology departments, could provide insights into how ambient lighting affects diagnostic performance across different environments. Furthermore, exploring the influence of lighting on a wider range of imaging modalities (e.g., MRI, functional imaging) could yield valuable insights into how lighting interacts with different types of imaging technology. Future studies could also consider comparing the performance of radiologists with varying levels of specialization (e.g., neuro-trained vs. non-neuro-trained radiologists) to determine if expertise modulates the effect of lighting conditions on diagnostic accuracy.

## Conclusion

In conclusion, our study provokes an important conversation on updating and expanding the literature on ambient lighting in radiology. As imaging technology continues to evolve, understanding how environmental factors such as lighting impact diagnostic performance will be crucial for optimizing radiologist efficiency and accuracy.

## Data Availability

The raw data supporting the conclusions of this article will be made available by the authors, without undue reservation.
